# A short note on HBV infection

**DOI:** 10.6026/97320630016505

**Published:** 2020-07-31

**Authors:** Aiswarya Muraleedharan, Giridharan Bupesh

**Affiliations:** 1 1Research and Development Wing, Central Research Laboratory, Sree Balaji Medical College and Hospital (SBMCH), BIHER, Chrompet, Chennai - 600044, India

**Keywords:** HBV, infection, update, diagnosis, treatment, prevention

## Abstract

HBV-related liver sickness or hepatocellular carcinoma is common worldwide. Therefore, it is of interest to document the current trends in hepatitis prevention, diagnosis, treatment and care.

## Description

Hepatitis B infection (HBV) is a global health issue. [1,2]. The presentation models for hepatitis B through immunizations is critical [3]. Hepatitis B surface antigen (HBsAg) is found on the surface of the infection by self-assembling, non-infectious circular or tubular particles [4]. The genome ORF encodes those three viral envelope proteins: large (L-), medium (M-), Also small (S-) surface antigen (HBsAg). A substitute ORF encodes precore, suggested as HBV e antigen (HBeAg), and the inside protein as the viral capsid. The ORF encodes the HBV X protein (HBx) [4]. The transcription factor-intervened guideline of HBV transcription has been described [5]. The HBeAg ORF encodes an endoplasmic reticulum (ER) [6]. HBeAg is fundamental for HBV replication [7]. The envelope proteins are incorporated in ER to form the transmembrane arrangement [8]. The luminal circle holds the major conformational epitope for HBsAg that is glycosylated on the S-protein moieties [9]. The adaptation of L is fundamental for capsids to assemble HBV virions [10]. These proteins make up the viral envelope [11]. SVPs are created with S-HBsAg, containing M-HBsAg and L-HBsAg [12]. The center protein in the HBV life cycle is a component of the capsid [13,14]. The inverse association between age and risk of chronic infection is responsible for the burden of morbidity and mortality to HBV [15]. At 6–12 months after infection, the immunoglobulin M antibodies to hepatitis B center antigen are imperceptible [16,17]. Immuno-suppressed persons might create reactivation from claiming HBV contamination resulting in hepatocellular carcinoma [18]. HBeAg, a marker for secondary viral action correlates with infectivity [19].

Low rate for HBsAg with medication in numerous patients is known [20,21].Transmission from a chronically infected woman with her newborn child throughout conveyance is productive [22]. Hepatitis B antibodies in immunization formulations utilizing two- four dose schedules are allowed in the United States [23]. Hepatitis B antibodies are immunogenic and post inoculation serologic demanding in the United States is observed under uncommon circumstances [24]. The structure of HBV surface antigen with correlation of subtype with amino acid sequence and location of the carbohydrate moiety is available [25]. Suspicions on viral safety in vaccine development are a concern [26]. Natural History and Clinical Consequences of Hepatitis B Virus Infection is a fear.[27]. The antivirals treatment in pregnant women is contraindicated in this case [28-30]. Rapid and sensitive assays for the determination of HBV genotypes and detection of HBV precore and core promoter variants are now available [14]. Thus, we document the current trends in hepatitis prevention, diagnosis, treatment and care in this short note.
